# Exploring a process-oriented way of working – a case study involving primary and specialised care

**DOI:** 10.1186/s12913-025-12435-z

**Published:** 2025-02-19

**Authors:** Hanna Odén Poulsen, Axel Ros, Jeffrey Braithwaite, Mattias Elg, Helena Kilander

**Affiliations:** 1 Jönköping Academy for Improvement of Health and Welfare, School of Health and Welfare, Jönköping University, Barnarpsgatan 39, 553 18 Jönköping, SE Sweden; 2 Region Jönköping County, Jönköping, Sweden; 3 Australian Institute of Health Innovation, Macquarie University, Sydney, Australia; 4 Department of Management and Engineering, Linköping University, Linköping, Sweden; 5 Department of Women’s and Children’s Health, Karolinska Institutet, Sweden and the WHO Collaborating Centre, Karolinska University Hospital, Stockholm, Sweden

**Keywords:** Clinical practice, Clinical routines, Health care organisation, Patient flow, Process orientation, Seamless care, Staff experiences, Working methodologies

## Abstract

**Background:**

Health care organisations have a long history of dividing work and tasks into decentralised functions and units by forming divisions and departments with delegated power. New ways of working, such as process-oriented approaches, have been called for to address challenges such as staffing shortages and resource constraints. There is limited understanding of the interplays that will occur in health care organisations during a shift from a traditional decentralised structure to a process-oriented approach. This study aims to explore the perceptions of health care staff and leaders when introducing a process-oriented routine.

**Method:**

We conducted interviews and focus groups with 29 participants in specialised and primary care in order to explore their experiences of a newly introduced routine regarding on-demand consultation, aimed at enhancing communication and patient coordination in a Swedish health care region. The participants included operating managers, schedulers and physicians. Data were analysed using reflexive thematic analysis in accordance with Braun & Clark’s guidance.

**Results:**

The findings encompass three main themes when introducing a process-oriented routine: *Creates a readiness to act, The critical role of trust for adopting on-demand consultation in everyday practice* and *Challenges associated with transformation*. The results show that health care staff and leaders are positive about the new way of working, but the readiness to act is challenged by issues of trust, as well as cultural components and structural factors such as experienced resource constraints.

**Conclusion:**

Our results underscore the need to consider not only organisational aspects but also social and individual relational factors when introducing a process-oriented way of work into a decentralised and complex health care system.

**Supplementary Information:**

The online version contains supplementary material available at 10.1186/s12913-025-12435-z.

## Introduction

Health care organisations globally have a long tradition of dividing work and tasks into decentralised functions and units [1–5]. Decentralisation is here defined as the formation of divisions and departments in health care, with delegated power [1]. Questions have been raised regarding how to get the health care system to work in cooperative harmony, and not as separated parts [4]. Some of the effects of the decentralised way of thinking and organising health care have been addressed from the perspective of patient outcomes (e.g. [6]), cooperation between professions, divisions and clinics [7–9], capacity to adopt innovations [10–12] and the need for developed leadership skills [13]. Health care is no exception to the need for developing organisational structures that build trust among citizens in public institutions [14]. The need is even more challenging in an era of reduced resources and rising demands for care and services.

One way to meet these challenges is to introduce new working methods in health care. Inspiration can be drawn from new business processes in manufacturing, or their quality improvement programmes, or both. A process-oriented way of working is defined in the manufacturing field as a logically-related series of standardised actions designed to achieve a desired outcome [15, 16]. The components involve collaborating, communicating, measuring and acting on results to achieve shared value and improve quality [15, 16]. This type of approach has given rise to the introduction in health care of process-oriented methodologies such as Lean [17–19]. Emphasising the creation of value from the patient's perspective is integral to concepts such as value-based care, co-production, and health care quality improvement [10, 20]. These initiatives also tend to emphasise the need for cross-sectional collaboration and communication. However, applying process orientation as defined in manufacturing in the context of health care raises questions due to the inherent complexity of the health care system. While such methods can bring about improvements, health care is a complex adaptive system with multiple stakeholders working in layered, intricate networks, with people independently and interdependently making decisions and taking actions in symbiotic relationships with each other [21]. Networks formed in a complex health care organisation based on professional and social needs of interactions are not necessarily the same as a process-orientation, taking into account the definition of process-orientation as a way of standardisation and improving quality [15, 16]. Complexity theory suggests that changes in a complex system—such as introducing more process-oriented ways of working—are unlikely to be linear or predictable. The outcomes of introducing this way of working in health care have proven difficult to interpret and the long-term effects are unknown [22, 23]. According to complexity theory, the direction and amount of change will therefore be hard to foresee and forecast [21]. In essence, in health care there is limited understanding of the interplays that will occur in health care organisations during a shift from a traditional decentralised structure to a process-oriented structure.

Swedish health care, like other health systems internationally, is facing considerable challenges, for example with shortages in resources and staffing. At the same time, the needs and demands for care are continuously increasing for multiple reasons [24]. There have been calls in Sweden for new ways of working, e. g. process-oriented work methodologies, and strategies for meeting these challenges [25, 26].

Within the context of this debate, we conducted a case study of a newly introduced process-based routine regarding enhanced communications between physicians in primary care and specialised care. The aim of our study was to examine how the introduction of this process-oriented way of working was perceived by the health care staff and leaders concerned.

## Methods

### Study design

Using a cross-sectional case study design we sought to integrate data from interviews and focus groups. This design was chosen so we could examine the events surrounding the workplace changes in their natural context and provide insights to support the sustainable introduction of process-oriented approaches elsewhere in Swedish health care and beyond. The study employed an inductive and descriptive approach since knowledge about the field is limited. The study was designed, performed and reported according to the Consolidated Criteria for Reporting Qualitative Research (COREQ) checklist [27].

### Context and setting

The context for the study is the Region Jönköping County, a health care region with 360 800 inhabitants in the south of Sweden. Region Jönköping County has three hospitals and 40 primary health care centres, integrated in one health care organisation with common management, where all healthcare facilities uses the same patient record information and referral system.

Region Jönköping County has approximately 10 000 employees, covering a range of social and health care services such as hospitals, primary health care centres, dental care, education and public transport. The county has a long tradition of structured quality improvement in health care with a systematic and academic approach [28–30]. The Region Jönköping County health care management group holds regular meetings with all the operating managers of the health care departments and the primary care centres in the health care region. Such meetings provide an arena for sharing common goals and for discussing needs for development and continuous improvement.

In Sweden, primary care is regarded as the foundation of community health, including both health care and health preventive measures. A national strategy for promoting integrated and coordinated high-quality care with the patient as an active co-creator has been ongoing since 2020.

### The case

**Fig. 1:**
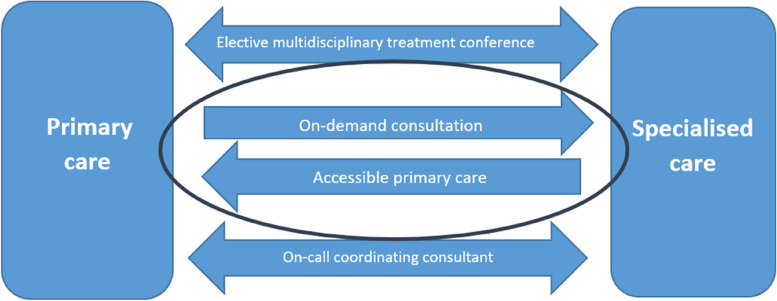
The four newly introduced tracks aiming to enhance communications between primary care and specialised care in the studied setting. This case study focuses on the two of the four tracks: On-demand consultation and Accessible primary care, which includes direct telephone communications between physicians

The case selected for this study is an initiative in Region Jönköping County aimed to meet the national strategy for promoting integrated and coordinated high-quality care. This initiative seeks to enhance efficiency in communications between primary care and specialised care. It takes its offspring in the national strategy and reflects changing demographics towards an elderly population, which results in increasingly complexity in patient care and the necessity for coordinated specialised care. The enhanced communication between primary care and specialised care aims to achieve better outcomes such as more patients receiving their full examinations and care in primary settings, facilitating efficient transfer to appropriate specialist departments at hospitals when specialised care is required, and ensuring safe transitions back to primary care once specialised care is no longer needed. The initiative for enhanced communications contained four tracks, see Fig. 1.

The studied tracks, on-demand consultation and accessible primary care, provide opportunities for physicians in both primary care and specialised care to adopt enhanced telephone communication during mutual patient consultations. At the time of the study, a change had been introduced that required both hospital specialist departments and primary health care centres to have a physician on duty and accessible by telephone for questions and consultations during office hours. All physicians at both primary health care centres and in specialised hospital departments could call each other for consultation. At the time of the study, there was no formal follow up regarding the stated patient outcome objectives of the initiative. There was, though, a recognized hypothesis among the initiators that enhanced telephone communications could reduce the amount of written referrals.

In order to develop and implement the new routines, a senior hospital clinician with extensive previous experience as an operating manager in a hospital department was appointed as project leader.

The authorisation of and commitment to the new way of working was provided by the Director of Health Care in Region Jönköping County. The on-demand consultative initiative was introduced in September 2022. Data collection was conducted during April 2023 – January 2024.

The first author (HOP) has worked in Region Jönköping County since 2014, being involved in first and second-line management until early 2023. This gives the author a pre-understanding of the context and setting and insights into issues surrounding the change and its unfolding dynamics.

### Sample and procedure

Theory-based and purposive sampling was applied [31] with the goal of deriving embedded perspectives and rich experiences from the case. The selection of participants focused on operating managers, schedulers and physicians, in order to best capture experiences from both front-line health care professionals and the first and second line of organisational management. The participants were recruited from both specialised hospital departments and primary health care locations. Departments and primary health care centres were purposively sampled based on input from the project leader regarding frequent routine usage. In total, four primary health care centres and three specialised hospital departments were invited to participate. Two primary health care centres and two specialised hospital departments agreed and were enrolled. Those declining cited lack of time and limited interest in the study topic from the professionals required for the study.

An invitation and information outline about how to participate in the study were provided by e-mail or orally at staff meetings. Operation managers and schedulers were invited to interviews. Physicians were invited to focus group discussions in order to capture interactions regarding experiences of both giving and receiving on-demand telephone consultation in their professional role, within the new routine. Once enrolled, all the participants were given more detailed oral and written information about the study and provided their written informed consent. Background data about both interview and focus group participants was collected. This included data regarding profession, years in the profession, age, and questions regarding the participants´ use of the routine. As background data, local status for each primary health care centre or department regarding number of written referrals before and after the introduction of the routine was also included, provided by HOP to the participants. The aims were to visualise the routine for the participants and again, as with the questions regarding use of the routine, to ensure that the first author and the respondents shared a common understanding about how the routine had affected the patient flow in the current setting.

Strategic documents describing the new system were reviewed to understand the enrolled sites and to establish a timeline for the introduction of the routine. Based on the document review, the first authors’ internal knowledge of the case, and discussion in the research group, a semi-structured interview guide was developed, using the same questions for both the interviews and focus groups (Appendix). The interview guide was tested in one single pilot interview with one participant working in Region Jönköping County who had extensive knowledge of both primary care and management. No major changes were considered necessary based on the pilot. Since no major changes were needed from the pilot interview, this information was treated as data and included in the material for analysis.

In total, nine interviews and five focus groups were conducted between April 2023 and January 2024, involving a total of 29 participants. The participants’ characteristics are presented in Table 1. Regarding use of the routine, primary care physicians mostly reported using it occasionally (one to three times a week) but noted they were rarely contacted as consultants (once a month or never). Physicians in specialised care confirmed that they did not often use the routine (once a month or never) but they frequently experienced being contacted in their role as on-demand consultants, often several times a day, when assigned to the role.

**Table 1:** Participants’ characteristics

	**n (%)**
*Profession*	
Operating managers	4 (14)
Schedulers	2 (7)
Physicians	23 (79)
*Gender*	
Male	9 (31)
Female	20 (69)
*Workplace*	
Primary care	17 (59)
Specialised care	12 (41)
Additional data	Mean (range)
Age	48 (26—65)
Years in profession	16 (1.5 – 36)
Years in current workplace	7 (0.5 – 35)

All interviews and focus groups, except one interview, were conducted face to face at the participants’ workplace during working hours. One interview was conducted through a video meeting, at the participant’s request. Focus groups each contained three to six participants. The median time for interviews was 30 min and for focus groups 50 min. The first author, HOP, led each session. Both interviews and focus groups began by clarifying the purpose of the study and by viewing the local status regarding numbers of written referrals for the respondents’ specific primary health care centre or department before and after the introduction of the routine. The first author then moved on to questions regarding the participants’ experiences and perceptions of the routine. Over time, reflection on the local status for written referrals were integrated into the questions, leading to a coherent flow in the conversation. Respondents in the interviews were offered the opportunity to review the transcripts as a member-checking measure for quality assurance, and three of them accepted the invitation. One participant responded with suggestions for adjustments to the text, which were accepted and the text documentation was corrected by the first author before the analysis. The focus groups discussions were verbally summarised by the first author at the end of the sessions, as a validation measure.

Interviews and focus group discussions were digitally recorded and transcribed verbatim. Names and places mentioned by the respondents were de-identified and the transcribed material was saved on a secure server accessible only by the first author. The background data, informed consent sheets, and the pseudonymisation key were stored in a locked space. After each interview and focus group, the first author wrote reflections on observations made and the flow of interactions during the session.

#### Data analysis

We conducted inductive qualitative analysis through reflexive thematic analysis based on Braun and Clark [32]. This approach allows for interpretation using the first authors´ pre-existing understanding of the context. We used the six phases described by Braun and Clark [32]. In phase one, the dataset with transcribed interviews and focus groups were read multiple times by the first author, for familiarisation. The written reflections made after every interview and focus group session were also reviewed, and new reflections were noted. In the second phase, the interviews and focus groups were coded by HOP. Coding was based on the research question *What are health care staffs´ experiences from the working routine with on-demand telephone consultation*. In phase three, the codes were arranged in candidate themes and subthemes around key concepts. These provisional themes were organised in a theme map using Excel (Microsoft Office Professional 2016), to enable analyses of possible connections, interconnections and contradictions within or between candidate themes. The theme map also served as a traceable documentation of steps in the analysis and development of the candidate themes. In phase four, data was arranged in 14 candidate themes by HOP and HK. The drafted candidate themes were phrased in English. These draft descriptions were discussed and re-worked iteratively with HOP, HK and AR, and later with all authors. In phase five, three main themes and ten subthemes were defined from the candidate themes, as a working model of the data. Throughout the data analysis, themes and subthemes were iteratively discussed with all authors. During the analytic phases the themes and subthemes were continuously checked against HOP’s written reflections made in connection with the interviews and focus groups. Thus, the study could draw on the pre-understanding of the context while at the same time ensuring that the interpretations stayed true to the data. During the analytic phases, HOP continuously wrote brief synopses of each theme, as a part of viewing and reflecting on the story told by the developing themes. In phase six, the final themes and subthemes were written up and agreed upon in the research group. Table 2 illustrates an example of an analytic trail and Table 3 presents the main themes and subthemes of the analysis.

**Table 2:** Example of analytic trail

Citation	Code	Subtheme	Theme
It feels like a very convenient way to get on with your work, I think. Some use the phone more, others like sending written referrals and awaiting an answer, and.. so on.	The way of working is person-dependent	Personal independence and organisational culture influence the way of working	**Challenges associated with transformation**

**Table 3:** Themes and subthemes

Theme	Subtheme
**Creates a readiness to act**	A motivation to change
	Creates a need to continue improving the system
	Facilitates individual and collegial learning in the professional role
**The critical role of trust for adopting on-demand consultation in everyday practice**	Trust in organisational capacity
	Trust in the way of working
	Collegial trust and cultural issue of power
	Need for shared perspectives
**Challenges associated with transformation**	Challenges with limited resources
	Specialisation in health care contributes to complexity
	Personal independence and organisational culture influence the way of working

### Ethical considerations

The Swedish Ethical Review Authority reviewed the study (Dnr 2023–01321-01). The key ethical considerations concerned maintaining internal confidentiality when conducting interviews and focus groups within a small, localised context. Information that could identify individuals or courses of action was excluded. It was also paramount that the research team, especially the first author, exercised sensitivity. It is well recognised that doing research in your own organisation as an insider requires an ethical and reflexive approach [33].

## Results

The qualitative analysis of interviews and focus groups regarding experiences from the routine on-demand consultation generated three main themes: **Creates a readiness to act**,** The critical role of trust for adopting on-demand consultation in everyday practice**, and** Challenges associated with transformation**. Subthemes were developed within each of these main themes, offering deeper insights, and these are detailed in Table 3.

### Creates a readiness to act

The results highlight a willingness and readiness in the organisation to act in, and on, the new way of working, and the participants expressed this in many corresponding views. The participants expressed a *motivation to change* involving a belief in positive effects for patients, a high expectation of being consulted in their professional role, and a hope that the amount of written referrals would decrease. The routine of on-demand consultation was perceived as being in alignment with other strategies in the organisation and also in line with how many participants experienced that they already worked. It was also indicated by the participants that the new routine was supporting other necessary changes both at their workplace and in the health care region, such as internal task-shifting for higher accessibility and the use of digital technology in communication with patients and other health care providers. One participant emphasised the importance and timeliness of these changes, stating:

*“ And this, we cannot deselect this, we really have to do it. I think it´s exciting and the timing is right.” Participant interview 1.*

A readiness to act also emerges through a *created need to continue improving the system.* The way of working was experienced to have evolved over time and it had given rise to new ideas about internal organisational mechanisms, and new routines. The participants identified areas of improvement outside the power of their own units regarding for example a need to adjust for economic effects and involving other competencies in the routine. The participants expressed the need for clear communication from senior management regarding resource allocation, prioritisation, and the monitoring of long-term objectives under the new routine. The participants reported a need and a will to continue with the new way of working but also a need to develop and adjust it to their current circumstances. That could entail, for example, clarification of responsibilities within the routine, and adjustments for differences in size and staffing between units.

*“We have solved the communications, and we have solved a lot of things in a very good way. But we need to solve the last pieces now …” Participant interview 5*

The results show that the created readiness to act also involves, and is related to, professional development such as learning opportunities. The participants stated that the routine *facilitated individual and collegial learning in the professional role*. They experienced enhanced communications with colleagues, and that the on-demand consultation led to learning opportunities that strengthened them in their professional role. It also created a willingness among the participants to take advantage of the learning revealed by the way of working, for example that consultation by telephone could reduce the need for written referrals, or that the synchronic on-demand consultation increased knowledge to such an extent that no consultation at all would be needed next time a similar event occurred.

*“It feels very good professionally to be able to help colleagues that call, give them some advice, thinking that you might educate them a bit with your advice.” Focus group 3*

#### The critical role of trust for adopting on-demand consultation in everyday practice

We turn to the central role of trust uncovered by the findings. The results show that trust is crucial for acceptance and use of the on-demand consultation routine in daily work. The participants stated the importance of *trust in organisational capacity* when introducing a new routine. This involved trust in the organisation’s ability to handle for example experiences and effects of increased workloads, and shifts in economic costs between specialised hospital departments and primary health care centres due to the new working routine. It also involved placing trust in the organisation’s capacity to increase the employers’ involvement in the new routines, and in the organisations ability to communicate the desired long-term goals with the new routine. The findings showed that the participants experienced an increased workload due to the new routine and that this negatively affected the quality of communications between units. The results indicated that the participants’ *trust in the way of working*, e. g. trust in the developed routine as something positive, declined when they experienced communicational inequities in their contact with other units; and the participants stated that they went back to their previous routines when they perceived substandard communication within the routine. There were diverging experiences across the interviews and focus groups regarding the professionals’ involvement in designing and introducing the routine. When the involvement experience was low – when the participants for example did not experience dialogue about the introduction of the routine – there was also a low level of trust in the organisational capacity and in the way of working as a routine. Many participants expressed a belief that on-demand consultation is positive for patients and the organisation. However, the participants also expressed the view that trust in the way of working was reduced due to the absence of a structured follow-up of the new routine. Thus, there were differing views, as shown by the following extracts:

*“I don´t think it /the routine/ has had any huge effect in our department.” Participant interview 2.*

*“… it generates a better flow, there are less detours and a faster flow and the ones that are referred to us are more relevant.” Participant interview 3.*

Alongside trust in the organisational capacity and trust in the way of working according to the routine, *collegial trust and the cultural issue of power* concerned trust between professionals in the organisation, and was a prominent feature. The participants valued the communications involved in seeking consultation by telephone; however, the quality of interpersonal professional communications was described as depending on the individual. It was also reported that interpersonal communication varied across units. The results showed that the manner in which individuals treated each other encompassed cultural elements that also impacted the level of trust inherent in the new routine. This concerned for example fears of receiving substandard communication when seeking consultation. The participants also reported the experience of their competence being questioned or not taken into account. A perceived cultural aspect of the cooperation between specialised care and primary care was described among the participants. This concerned for example experiences of inquiries from specialised care were given precedence over those from primary care. Participants from primary care also described an experience of shifts in tasks and pressure on economic costs that led to a sense of “working on behalf of others”. This can be exemplified by this quotation from a primary care physician:

*“..and then I called and wanted the patient /*to be attended to*/ fast, and this colleague he –- I felt like he did not really believe me, that this could not be /a specific medical diagnosis/.” Focus group 1.*

The results also indicate a lack of trust, not just between some individuals but also between units. The participants expressed concerns, for example, that others would not use the routine in “the right way”. The participants considered other units outside their own to have less capacity in their internal structures or a reduced ability to adjust internal resources in order to meet the needs of the routine. That said, the experience of most participants was that the way of working was mostly applied as intended. This was further highlighted in the last subtheme regarding the critical role of trust – a *need for shared perspectives* – that was evident across the interviews and focus groups*.* When a lack of shared perspectives was reported, this gave rise for example to feelings of being misunderstood in communication, experiences that the answers in consultation did not match the needs, or uncertainty about whether the consultation given was appreciated or even used. Lack of shared perspectives also gave rise to uncertainties regarding practical aspects of the routine, for example, responsibilities when referring patients to other units, documentation and economics. Some participants indicated that they experienced low levels of insight into how others elsewhere in the system applied the new routine, what others thought about it and how it affected their workloads. Some participants stated that they knew very little about other’s circumstances, especially between units, and felt that if knowledge was shared and widely available the way of working with on-demand consultation could be improved further. For example:

*“You know that many departments have morning meetings somewhere between eight and nine, so maybe it´s not very a good time to call all the time. I think so but I don´t know. But maybe that time is* *really good? /…/ It´s only my guess really. I don´t know how many resources they put into this when acting as on-demand consultants.” Focus group 2.*

#### Challenges associated with transformation

The participants raised issues that posed barriers to the change. *Challenges with limited resources* were specifically expressed by the participants in specialised care but were also reported among primary care participants. Such challenges became apparent in reports of reduced accessibility to on-demand consultation when resources in specialised care were perceived to be scarce. Those working in the new routine experienced an increased workload in specialised care and the participants expressed difficulties in allocating resources appropriately. They also expressed worries and uncertainties regarding resources in the long term. The variety of inquiries and the large number of questions created challenges in planning the daily work tasks, and gave rise to clinicians needing to prioritise. This quote exemplifies this:

*“I´m still very positive about the project and the process. What has been hard is the heavy workload for the physicians in the department, which has really increased regarding telephone calls. So it´s, yes, I´m ambivalent in that questions because you also care about your employees ‘ working environment.” Participant interview 2.*

The primary care participants did not say they had to adjust internal resources to handle the new way of working. These differential findings across the two groups illuminate diverging experiences regarding the need for internal adjustments in resources to manage workloads under the new routine – some parts of the system experienced a need to make major changes while some saw no need for change. Furthermore, the high degree of specialisation in today’s modern health care delivery systems was seen by the participants as giving rise to an extended need for consultation, and there was a perception that *specialisation in health care contributes to complexity.* For example:

“*Then the consultant, the on-demand consultant, couldn’t answer about- – But call this /specialist/-physician or no, he could not answer either but call this /specialist/-physician. No, he could not answer but call someone – next. And, of course, if you are so sub-specialised, then it´s hard to get a good flow in the questions. And it´s hard to know for the one calling when to bypass the on-demand consultant and call the super-specialist instead, and when not to.” Participant interview 3.*

Other tasks, such as educational assignments and in-house consultation, were perceived as already contributing to the daily complexity faced by the participants. The participants made the point that prioritising was an internal activity undertaken to handle this complexity, and that prioritising by definition affected other areas in their own unit or across other parts of the system.

The results also revealed opinions from the participants that *personal independence and organisational culture influence the way of working*. For example, there were person-dependent variations in using the routine and every unit acted to solve the practical routines inherent in the way of working in their own way. There was an awareness among the participants of being a part of a complex health care organisation and that such an organisation is by tradition difficult to change. The participants did express an awareness that local culture at the units affected how the way of working was introduced, and that, for it to prevail, the routine had to become a part of a changed culture. An evocative extract explains this:

*“But we are still—when I´m the on-demand consultant I still take care of written referrals as well, I can´t let it go /small laughter/ since we have worked like that for many years and it´s a collegial thing to unburden each other. No, so- that’s just how we work.” Participant interview 4.*

## Discussion

### Main findings

Our qualitative, cross-sectional case study enabled us to explore experiences of the introduction of the process-oriented routine on-demand consultation from the perspective of staff and leaders in primary care and specialised care, who were working in the health care system on a daily basis. The findings contribute to the understanding of the interplays that occur in a shift from a traditional decentralised structure to a process-oriented one, and important factors for a sustainable introduction of new ways of working in the perspectives of frontline staff and their leaders. The results show that when introducing this process-oriented routine, health care staff and leaders share congruent experiences regarding a readiness to act. However, this is challenged by issues of trust, as well as cultural components and structural factors like resources. At the time for the study, no structured follow-up had been conducted concerning the stated patient outcomes objectives of the initiative of enhanced efficiency in communication. Furthermore, there were no structural follow-ups regarding the hypothesis that increased telephone consultation would affect the amount of written referrals. Consequently, our results are discussed solely based on the participants´ described perceptions. As shown in the results, perceptions amongst participants varied regarding whether the initiative with on-demand consultation and accessible primary care truly impacted patient outcomes.

Our results suggest that health care staff and leaders are willing to contribute to a new way of working. They expect having opportunities to influence, and that the routine is governed. This corresponds with the findings related to introducing process-oriented structures in a study by Trankle et al., which describes experiences of the need for anchoring and developing new models as crucial to sustainability [34].

Different aspects of trust are highlighted in our study as essential for adopting the new way of working, and also the need for shared perspectives to create this trust. The different aspects of trust concerns trust in the organisation, trust in the way of working with on-demand consultation as a routine itself, and trust between different parts and individuals in the health care system. Trust in both individuals and in the organisations has previously been shown to be an indicator for the successful introduction of a chain of care [35], and important for building partnerships [36], and our results indicate it is also a crucial factor in introducing a process-oriented way of working.

In our study, the participants’ experience of how their professional role and knowledge are being taken into account seems to be crucial aspects influencing trust. This indicates that shared understanding not only of the way of working as a practical routine, but also of each other's working conditions as professionals and individuals in the system is essential. This is exemplified by the result regarding the importance of experiencing equal and professional communication in contacts with other units in order to use the routine. Social structures and social identity are known to be factors to take into account when understanding health care organisations [37]. The social identity can be strongly connected to (inter)group relations in health care [38], and can in itself contribute to working in silos. This suggests that when introducing a process-oriented way of working, the need for shared perspectives among involved parties must also include an understanding of a new constellation of group identity to bridge not only organisational silos but also social silos. At the same time, changing individual and professional perceptions of meaning in favour of shared understanding of an event is demanding [39]. In our study, the call for enhanced communication about long-term goals and for a structural follow-up reflects the need to ensure that the changes in actions, perspectives, and even social identity are truly justified when health care staff need to shift from task-based roles to a more cross-functional collaborative approach.

The results show that there was an awareness among the participants of being part of a complex system. At the same time, they expressed a need to adapt the routine to specific internal conditions, yielding unique arrangements in internal structures that challenged the new way of working. This corresponds to health care being a complex adaptive system where front-line professionals have a considerable degree of autonomy [21]. Although there are formal requirements, the complex adaptive system tends to act as a self-regulating ecosystem [40] making structured follow-ups of this kind of changes difficult. The health care organisation seems to have to balance both the expressed need for uniqueness with the need for structured follow-ups, to create trust and sustainability in the introduction of new ways of working.

Challenges with scarce resources, in our study involving both staffing and economic resources, are identified as important. In both our study and others [8, 34], limited resources are raised as obstacles to introducing new ways of working. Staff and leaders in our study expressed experiences of the routine actually requiring more resources, and this can be seen as contradictory to the national aim of introducing new routines to make the care system more efficient. This indicates that efforts should be made to create shared perspectives not only among staff and leaders working together in a new routine but also between staff and organisational top management regarding the intended outcomes of a new way of working. For the sustainable introduction of process-oriented routines, success factors have been shown to include alignment with the organization's norms, values, and management style [35], as well as fostering a shared understanding between clinicians and managers [41]. Our results indicate that clarification of, for example, how to handle experiences of scarce resources could be one way of creating this shared understanding.

Other studies [8, 42, 43] describe experiences of obstacles to introducing process-oriented ways of working, such as different IT systems for information, and employers not belonging to the same organisation. Our study did not show results suggesting that IT or employee status raised barriers, indicating that these areas are not considered obstacles in the studied setting where all health care facilities have the same patient record information and referral system and employees are organised under the same health care management group. But regardless of that, the participants still raised other aspects challenging the transformation. This underlines the need to address technical, organisational, and social domains in transformations [44], indicating that all these aspects are also necessary for the sustainable introduction of process-oriented approaches in health care.

### Strengths and limitations

A major strength of this study is the empirical design, which allows us to examine the new working routine in its natural context. The study provides an insight into the daily and practical work with new ways of working in the complex health care system, a dynamic interplay difficult to capture theoretically. In order to conceptualise how a process orientation is affected by complex and decentralised health care, a number of routines, processes, and settings may be of interest to investigate. We are aware that this limited case study may only provide a fraction of the potential experiences and perceptions of health care staff and leaders. We are aware that only participants involved in the routine of on-demand consultation, both physicians working according to the routine and their operating managers and schedulers, were included in the study, and that aspects from those who have not adopted the new way of working were not included. We also acknowledge that this study was conducted at an early stage of the introduction of the routine, and that such routines are likely to evolve and be refined over time, with the described experiences therefore representing the event at a specific point in time. Nonetheless, we view this cross-sectional case study as a suitable way to start exploring and establishing a qualitative baseline in the field. Case study approaches are suitable for exploring professionals' attitudes and experiences in a real-life context, and can unpack the dynamics of changes [45, 46]. Our case involves clearly delimited functions in health care and a routine with a clear aim, both nationally and locally, to create integrated and coordinated high-quality care by working across boundaries. The routine is likely to impact everyday work in a wide range, which is also confirmed by the results. We are aware of the context-dependent aspects of case studies [47, 48]. For example, the result in this study regarding on-demand consultation being experienced by the participants as in alignment with organisational strategies may be an effect of Region Jönköping County having a long history of structured quality improvement with an academic approach and may not be as evident in settings operating under other circumstances. However, we believe that the results from this study are applicable elsewhere in Swedish health care and beyond if contextual factors are taken into account. A limitation of our study is not including the experiences of patients' as the end-point customers of the new routine.

The validity of the study [49] is strengthened by the prolonged data collection, incorporating aspects from both specialised and primary care, front-line and first- and second-line management. Using member-checking and incorporating the participants' feedback further strengthens the study, as does using a pilot interview to validate questions and interview techniques. Reliability was sought in describing the steps in the thematic reflexive analysis, providing examples of an analytic trail and linking results to transcripts by presenting quotes to illustrate subthemes. We are aware that the participants might have known the first author by name before being enrolled in the study, but we do not expect any bias to affect the study because of this. We consider it a strength that the first author is an insider in the organisation, thereby contributing with pre-understanding in the design of the study and interpretation of the results [50]. The analytic method used, reflexive thematic analysis, allows the study to draw on pre-understanding but demands an awareness of how this can affect interpretations [32]. Written reflections, self-awareness of possible impacts, and the research group's involvement in coding and analysis were strategies used to ensure that interpretations and results were grounded in data.

### Implications and future research

Our results may help guide health care staff, leaders and decision-makers when introducing new ways of working in health care settings. Our study highlights the importance of building trust in and between involved stakeholders. Further research is needed regarding how health care staff act in organisational transformations while being part of the complex health care system, in order to explore factors affecting the perceptions of trust. Research is also needed on the patients' and next of kin's point of view regarding how organisational structures and new ways of working affect their care process and their lived experiences.

## Conclusion

Health care staff and leaders expressed a positive attitude toward the introduced process-oriented routine of on-demand consultation, but a critical role of trust is associated with sustainable transformation; there are challenges concerning the experience of scarce resources, health care specialisation, personal independence as well as organisational culture. Our results underscore the need to consider not only organisational but also social and individual relational aspects when introducing a process-oriented way of work into a decentralised and complex health care system.

## Supplementary Information

Supplementary Material 1. Appendix—Interview questions

## Data Availability

All data generated and analysed during the current study are not publicly available due to the confidential nature of participant transcript data but are available from the corresponding author on reasonable request.
