# Supplementary material for: Exploring a process-oriented way of working – a case study involving primary and specialised care
Source: BMC Health Serv Res. 2025 Feb 19;25:282. doi: 10.1186/s12913-025-12435-z (PMC11841351; doi:10.1186/s12913-025-12435-z)
Supplement: Supplementary file 1 — Supplementary Material 1. Appendix—Interview questions [file 12913_2025_12435_MOESM1_ESM.docx]

Appendix - Interview questions

1. How did you first receive information about the On-demand-consultation routine?

2. What were your initial thoughts about this way of working?
Possible follow-up questions: Pros? Cons? Have your thoughts changed over time?

3. How was the routine implemented in your clinic/department? What has worked well and why? What has been problematic and why? (For operating managers: Have you been able to impact or adjust the routine to fit your circumstances?)

4. Have you found that the routine impacts the patient?
Possible follow-up questions: In a positive or negative way? Can you provide an example?

5. Have you found the routine impacting your clinic/department or your way of working in general? Possible follow-up questions: In a positive or negative way? Pros? Cons? Can you provide an example? (For operating managers: Has the way of working had any impact on the way you lead and govern your clinic/department?)

6. What are your reflections regarding the local data /of referrals/ for your clinic/department?

7. What are your thoughts on the introduction of process-oriented ways of working, such as on-demand consultation, in healthcare?

8. Is there anything else you would like to add or share regarding the introduction of the on-demand consultation routine that we haven't addressed? Or regarding any other process-oriented way of working that you have encountered?
